# SoyFN: a knowledge database of soybean functional networks

**DOI:** 10.1093/database/bau019

**Published:** 2014-03-10

**Authors:** Yungang Xu, Maozu Guo, Xiaoyan Liu, Chunyu Wang, Yang Liu

**Affiliations:** ^1^School of Computer Science and Technology, Harbin Institute of Technology, Harbin 150001, P.R. China and ^2^School of Life Science and Technology, Harbin Institute of Technology, Harbin 150001, P.R. China

## Abstract

Many databases for soybean genomic analysis have been built and made publicly available, but few of them contain knowledge specifically targeting the omics-level gene–gene, gene–microRNA (miRNA) and miRNA–miRNA interactions. Here, we present SoyFN, a knowledge database of soybean functional gene networks and miRNA functional networks. SoyFN provides user-friendly interfaces to retrieve, visualize, analyze and download the functional networks of soybean genes and miRNAs. In addition, it incorporates much information about KEGG pathways, gene ontology annotations and 3′-UTR sequences as well as many useful tools including SoySearch, ID mapping, Genome Browser, eFP Browser and promoter motif scan. SoyFN is a schema-free database that can be accessed as a Web service from any modern programming language using a simple Hypertext Transfer Protocol call. The Web site is implemented in Java, JavaScript, PHP, HTML and Apache, with all major browsers supported. We anticipate that this database will be useful for members of research communities both in soybean experimental science and bioinformatics.

**Database URL:** http://nclab.hit.edu.cn/SoyFN

## Introduction

Soybean (*Glycine max*), an important domesticated species originating in China, constitutes a major source of edible oils and high-quality plant proteins worldwide. In spite of its complex genome as a consequence of an ancient tetraploidization, platforms for map-based, sequence-based, comparative and functional genomics have been well developed in the past decade. Thus, rich repertoires of genomic tools and resources are available, which have been influencing the soybean genomic improvement. Several databases for soybean genomic analysis have been built and made publicly available, such as SoyGD (1), SoyXpress (2), SoyBase (3), SFGD (http://bioinformatics.cau.edu.cn/SFGD/), SoyDB (4) and SoyKB (5), containing a variety of information, such as soybean genome sequences, bacterial artificial chromosome, expressed sequence tags and some useful tools including genome browsers, BLAST searching and pathway searching. Even so, these databases only contain general annotations for the soybean genome, instead of knowledge specifically targeting the genome-wide gene–gene, gene–miRNA and miRNA–miRNA interactions. However, similar accomplishments have been achieved in many model organisms. First, functional gene networks (FGN) have been successfully constructed and made available in yeast (*Saccharomyces cerevisiae*) (6), nematode (*Caenorhabditis elegans*) (7, 8), Arabidopsis (*Arabidopsis thaliana*) (9, 10), rice (*Oryza sativa*) (11), mouse (*Mus musculus*) (12–14) and even the human species (*Homo Sapiens*) (15). Some of these FGNs are available at http://www.functionalnet.org. Second, several elegant experiments have been carried out, unraveling intriguing microRNA (miRNA) interactions (Enright *et al.*, 2004; Krek *et al.*, 2005; Shalgi *et al.*, 2007; Chen *et al.*, 2010; Xu *et al.*, 2011), which usher in new insights into miRNA that focus on network rather than on individual interaction. Although SFGD contains knowledge about the networks of genes and miRNAs, it only covers 23 267 genes and 193 miRNAs, far less than the current numbers of genes deposited in EnsemblPlants (54 174 protein-coding genes, JGI-Glyma-1.1) (16) and miRNAs in miRBase (555 mature miRNAs generated from 506 hairpin precursors, release 19) (17). Therefore, it is necessary to provide a new database for retrieving and analyzing gene–gene, miRNA–miRNA and gene–miRNA interactions on the genome- and/or microRNome-level.

Here, we present SoyFN, a database of soybean functional gene networks (SoyFGNs) and miRNA functional networks (SoymiRFNs) partially based on our previously published work (18, 19). SoyFN is a schema-free database that can be accessed as a Web service from any modern programming language using a simple Hypertext Transfer Protocol (HTTP) call. SoyFN provides interfaces to freely retrieve, visualize, analyze and download the functional network of soybean genes and miRNAs. The SoyFN Web site can also be used to access the integrative information about genome context provided by genome browser, participated pathways by Kyoto Encyclopedia of Genes and Genomes (KEGG), Gene ontology annotations (GOA) by UniProtKB and EnsemblPlants, etc., as well as to convert gene ID between different identifiers and to compute gene (or GO term) functional (or semantic) similarity by using our previously proposed method (19).

## Database construction and data description

### Construction information

**Figure 1.**
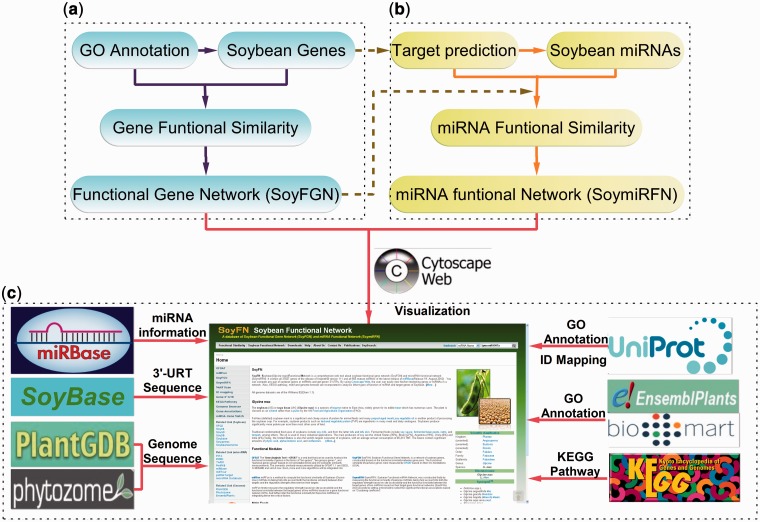
Procedure for SoyFN construction. (**a**) Inferring soybean GFSAT and SoyFGNs. (**b**) Inferring soybean miRNA functional similarity and SoymiRFNs. (**c**) Incorporating other publicly accessed databases and tools to support and enhance the analyses of soybean genomic and microRNomic interactome.

SoyFN was designed to store, retrieve, visualize and analyze soybean function network of genes and miRNAs in an omics level. The procedure for SoyFN construction is shown in Figure 1. Briefly, SoyFN construction comprises three parts: (i) measuring gene functional similarity based on GOA, which was implemented as a species-free gene functional similarity analysis tool (GFSAT) (19), inferring the SoyFGN based on the cluster coefficient threshold selection (to be published), which was visualized by Cytoscape Web (Figure 1a); (ii) measuring soybean miRNA functional similarity based on consideration of both the accessibility between miRNA and its target genes and the interactive information between target genes in a whole gene functional network (SoyFGN) (18), inferring the SoymiRFN based on the cluster coefficient threshold selection (18), which was visualized by Cytoscape Web (Figure 1b); and (iii) incorporating many other publicly accessed databases and tools to support and enhance the analyses of the soybean genomic and microRNomic interactome (Figure 1c).

### Data description

Four types of original data sources were used to implement current version of SoyFN: (i) GO terms and their relationship information were downloaded from The Gene Ontology Web site (version V1.1.3499, http://www.geneontology.org/); (ii) GOA of soybean genes were downloaded from both UniProtKB (version v111, http://www.ebi.ac.uk/GOA) and EnsemblPlants/BioMart (version V1.0, JGI-Glyma-1.1, http://plants.ensembl.org/index.html); (iii) genome sequences and 3′-UTR (Untranslated Region) sequences were downloaded from SoyBase (http://soybase.org/), PlantGDB (http://www.plantgdb.org/) and Phytozome (http://www.phytozome.net/); and (iv) miRNA sequences were downloaded from miRBase (Release 19, http://www.mirbase.org/). A detailed statistics of these data sources are shown in Supplementary Table 1 and fully given on the Web page named ‘Statistics’, which can be directed to by clicking the top navigation bar at ‘About Us/Statistics’. All data available can be downloaded from the download page.

SoyFGN and SoymiRFN are two most important extended data sources that SoyFN provides for users. SoyFGN provides the first version of three SoyFGNs, including 25 835, 28 833 and 14 136 genes in SoyFGN-BP, SoyFGN-MF and SoyFGN-CC, which account for ∼70% (33 807) of the 54 174 soybean genes recorded by EnsemblPlants (version 18, April 2013). The availability of the second version of SoyFGNs covering all 54 174 genes is under way. Based on a novel approach to measuring the functional similarity of miRNAs, considering both their target site accessibility (20) and the topology of target gene functional network (SoyFGN), SoymiRFN provides four miRNA functional networks in Biological Process (BP), Molecular Function (MF), Cellular Component (CC) and Integration, which covers 462, 454, 512 and 472 miRNAs, respectively (18). A detailed topological properties of SoyFGNs and SoymiRFNs are listed in Supplementary Table S2.

### Implementation

**Figure 2.**
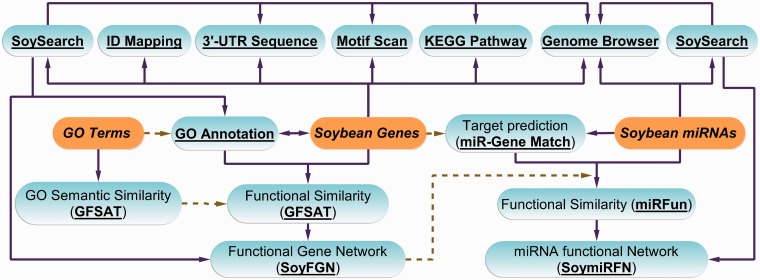
Architecture of SoyFN database. Solid lines mean that the user can choose the analysis path according to results obtained at each previous step. Dashed lines mean that the results of the previous steps will be used in the next steps as source data. Italic words with highlighted background represent the most original data sources used by SoyFN. The underlined bold words represent the main functional modules and useful tools can be implemented on SoyFN.

SoyFN is a browser-independent Web database built using Java, JavaScript, PHP and HTML and implemented in Apache to retrieve, visualize and analyze the system-level interactions of soybean genes and miRNAs. It also incorporates many related useful tools to provide more comprehensive information about soybean genome and microRNome. The architecture of SoyFN is shown in Figure 2. By means of the detailed instructions on SoyFN, user can easily run each functional module or tool. SoyFGN and SoymiRFN provide the interfaces to retrieve, visualize and analyze the networks of a list of genes and miRNAs, embedding and interacting with Cytoscape Web (21). GFSAT is used to compute the semantic similarity of GO terms and the functional similarity of genes, supporting three methods and > 30 species. miRFun is used to compute the functional similarity of soybean miRNAs based on the topological information of their target gene network (SoyFGNs) and the binding accessibility (20) between miRNA and its targets. SoySearch is used to perform an integrated search by one gene or miRNA on many databases provided by SoyFN. All query results and data sources can be freely downloaded according to user’s needs. In addition, many useful tools, including ID mapping, Motif scan, KEGG pathway and Genome Browser are available for users to get more related information of the genes or miRNAs from the publicly accessed third-party databases. Moreover, there are friendly interactive query interfaces between all functional modules and tools (Figure 2).

### Database use and access

This section, as use cases, we describe two of the main implementations of SoyFN to show a miniature that SoyFN provides users with the integrative information of soybean genes and miRNAs, which will facilitate the researches for members of research communities both in soybean experimental science and bioinformatics. One is to using SoyFGN to retrieve, visualize and analyze SoyFGN, and the other is to use SoySearch to investigate all available information of an individual soybean gene or miRNA that was released by the current version of SoyFN.

### Retrieve, visualize and analyze soybean functional networks of genes and miRNAs

**Figure 3.**
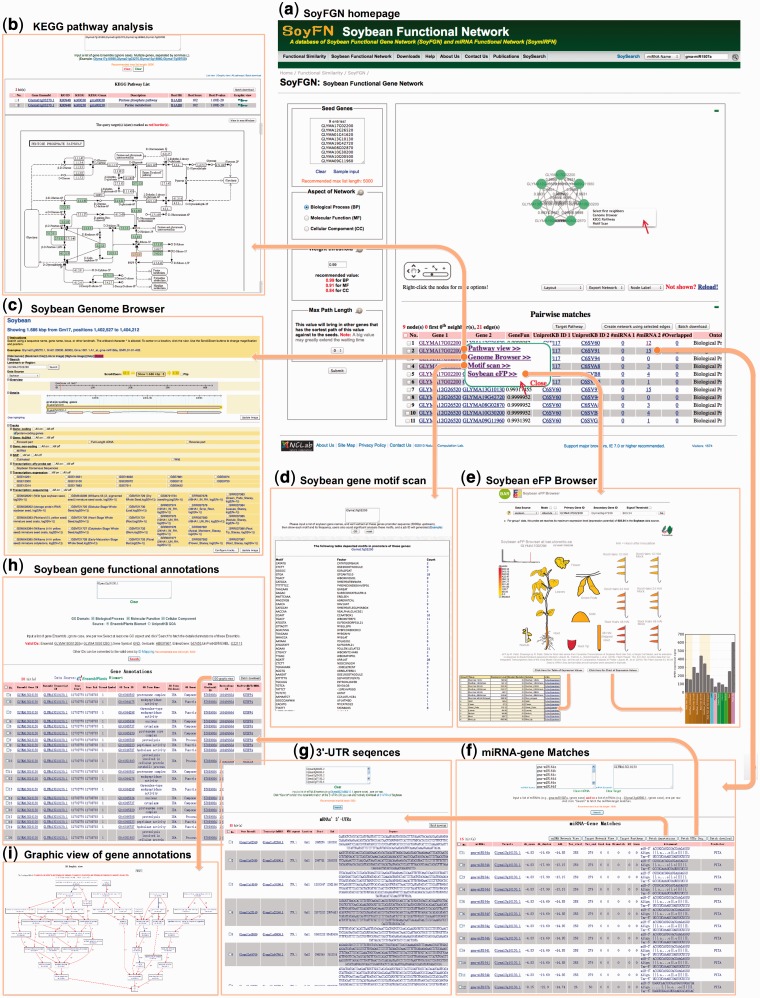
A workflow to retrieve, visualize and analyze the SoyFGN using SoyFGN. (**a**) The home page of SoyFGN and the results by submitting the sample genes with the default parameter settings. (**b**) KEGG pathway analysis of the selected genes. (**c**) Showing a specific gene and its genome context in Genome Browser. (**d**) Retrieving the gene’s promoter motifs from PLACE (http://www.dna.affrc.go.jp/PLACE/). (**e**) Displaying the expression profiles of a specific gene in different tissues or organs by ‘eFP’. (**f**) Matches between a specific list of genes and miRNAs, which provides the interaction between SoyFGN and SoymiRFN. (**g**) Getting the 3′-UTR sequences of genes. (**h**) GO function enrichment analysis of genes. (**i**) Graphic view of the enriched GO terms.

SoyFGN and SoymiRFN provide the interfaces to retrieve, visualize and analyze the network of a list of soybean genes and miRNAs. Here, we use SoyFGN to retrieve, visualize and analyze SoyFGN, of which the workflow can be an example of using SoymiRFN to investigate the miRNA functional network in spite of some differences between them. In this case, we use the sample genes (click ‘Sample input’ under the input box) and default parameter settings; more detailed instructions for parameter setting can be found at ‘Help/Functional Gene Network’. After submitting, the network visualized by Cytoscape Web will be shown at the top of the right panel of SoyFGN page, followed by the pair-wise genes listed in an overview table presented later (Figure 3a). Interacting with Cytoscape Web, users can zoom into (or out of) the network view, change the layout, switch the node labels and export the visualization in multiple formats. When mouse-over a gene ID in the table or right-clicking a node in the network, a pop-up panel will show with more options to direct users to different pages for further analyses (indicated by two red arrows in Figure 3a). Clicking the ‘Pathway view’ link in pop-up or the ‘Target Pathway’ button above the table, users will be directed to a new page analyzing the KEGG pathway annotations of the selected genes (Figure 3b). The found genes and corresponding KEGG annotations will be listed in the table with one entry per row. Clicking the KEGG icon of the last cell of a row will show the graphic view of the pathway, in which the query genes are highlighted by red rectangles (Figure 3b). When the ‘Genome Browser’ link is clicked, the gene and its genome context will be shown in a genome browser page (Figure 3c). Clicking the ‘Motif scan’ link, users will open a new page to retrieve the gene’s promoter motifs from PLACE (http://www.dna.affrc.go.jp/PLACE/) (Figure 3d). eFP Browser provides the ‘electronic fluorescent pictographic (eFP)’ representations of the gene of interest’s expression patterns in different soybean tissues or organs. By clicking the ‘Soybean eFP’ link, users will open a new page to display the expression profiles of a specific gene in an interactive pictographic view (shown in Figure 3e). Users can switch between table and chart view of expression values of this gene (shown as embed sub images in Figure 3e) by clicking the two buttons below the pictography, and can alternatively get the detailed expression data sources from PLEXdb (http://www.plexdb.org/index.php) by clicking any part of the soybean organs in the pictography.

Another important information on SoyFGN page (Figure 3a) are the three columns named ‘#miRNA 1’, ‘#miRNA 2’ and ‘#Overlapped’ in the overview table, which represent, respectively, the number of miRNAs predicted to regulate the ‘Gene 1’, ‘Gene 2’ and the number of miRNAs predicted to co-regulate both of these two genes. Clicking the underlined number, a new page named ‘miRNA-Target Match’ (Figure 3f) will be opened to display the detailed matching information between the genes and their regulating miRNAs predicted by three methods (18). Using ‘miRNA-Target Match’, SoyFN provides an interaction between SoyFGN and SoymiRFN. Users can, respectively, generate a gene network and miRNA network using a list of matched genes and miRNAs of interest by clicking the two buttons above the table (shown in Figure 3f). The ‘Fetch UTR Seq.’ button is used to get the 3′-UTR sequences of the selected genes (Figure 3g). The ‘Fetch Annotations’ button is used to analyze the GOA of the selected genes from two different annotation databases: UniProt-GOA and the BioMart of EnsemblPlants (Figure 3h). Using this page, user can investigate which functions the selected genes are enriched in and generated a graphic view of the annotated GO terms by simply clicking the ‘GO graphic view’ button above the table (Figure 3h and i). All whole query results aforementioned can be downloaded to local computers from the download buttons above the overview tables.

### Search a specific miRNA or gene using SoySearch

**Figure 4.**
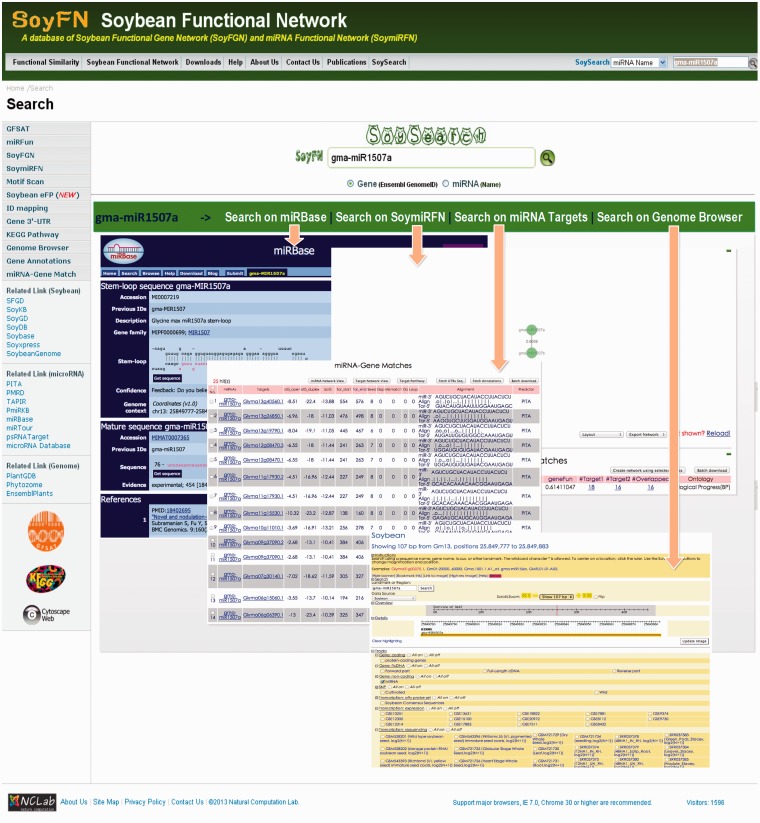
A use case to retrieve an miRNA in SoyFN using SoySearch. Four types of information about the specific miRNA will be returned and shown below the input box. Users can click the tabs to switch between them and will be redirected from SoySearch to the homepage of each functional module to implement more detailed analyses by clicking ‘More on…’ option in each tab.

The SoySearch toolkit is developed to acquire the collected information of a specific gene or miRNA of interest within and outside the SoyFN. Here, we only search a sample miRNA (gma-miR1507a) to show the information can be acquired by using SoySearch, of which the workflow is also suitable for searching a gene. User can use the SoySearch toolbar on every page to start a new search or click the ‘SoySearch’ link to open the SoySearch homepage (Figure 4). Current release of SoySearch only support Ensemble Genome gene ID and the name of mature miRNA. Any other kinds of gene IDs can be mapped to Ensemble Genome IDs using the ‘ID mapping’ tool. When the miRNA is submitted to SoySearch, the results will be shown below the input box. Four types of information available for a specific miRNA in SoyFN will be shown in the result panel one at a time: (i) the published miRNA sequence and annotation provided directly by searching on miRBase, (ii) the direct neighbors of the miRNA in four SoymiRFNs provided by SoymiRFN, (iii) the predicted target genes of this miRNA provided by miRNA-Target Match and (iv) the genomic information of the miRNA provided by Genome Browser (shown as four embed sub images in Figure 4). User can switch between them by simply clicking the corresponding tabs. Clicking ‘More on…’ option in each tab, users will be redirected from SoySearch to the homepage of each functional module to implement more detailed analyses.

## Conclusion

SoyFN is the first Web database providing comprehensive information on soybean gene–gene, miRNA–miRNA and gene–miRNA interactions in omics level. It is a schema-free database that can be accessed as a Web service from any modern programming language using a simple HTTP call. Although SFGD contains knowledge about the networks of soybean genes and miRNAs, it, first, only covers a small number of genes and miRNAs, far less than that deposited in public databases. Second, in SFGD, the relations between genes and miRNAs were inferred from limited co-expression profiles of their coding genes. While in SoyFN, all genes and miRNAs are connected on the notion of their functional similarities, which are more intuitive to reflect the associations between genes and miRNAs in functionality. Additionally, SoyFGN embeds much information including KEGG pathways, GOA and 3′-UTR sequences, as well as many useful tools including SoySearch, ID mapping, Genome Browser, eFP Browser and promoter motif scan to provide more comprehensive information about soybean genome and microRNome.

## Supplementary Data

Supplementary data are available at *Database* Online.

## Funding

Natural Science Foundation of China (61172098, 61271346 and 91335112) and the Specialized Research Fund for the Doctoral Program of Higher Education of China (20112302110040). Funding for open access charge: 61172098.

*Conflict of interest.* None declared.
